# 胸腺素β10在肺癌细胞中促进VEGF-C表达机制研究

**DOI:** 10.3779/j.issn.1009-3419.2014.05.03

**Published:** 2014-05-20

**Authors:** 紫璇 李, 连悦 曲, 红珊 钟, 克 徐, 雪杉 邱

**Affiliations:** 1 110001 沈阳，中国医科大学附属第一医院病理科，基础医学院病理学教研室 Department of Pathology, the First Affiliated Hospital of China Medical University and College of Basic Medical Sciences, China Medical University, Shenyang 110001, China; 2 110001 沈阳，中国医科大学附属第一医院放射科，辽宁省影像诊断与介入治疗重点实验室 Department of Radiology and Key Laboratory of Diagnostic Imaging and Interventional Radiology of Liaoning Province, the First Affiliated Hospital of China Medical University, Shenyang 110001, China; 3 110001 沈阳，中国医科大学附属第一医院药学部 Department of Pharmacy, the First Affiliated Hospital of China Medical University, Shenyang 110001, China

**Keywords:** 肺肿瘤, Tβ10, 磷酸化蛋白激酶B, 血管内皮生长因子C, Lung neoplasms, Thymosin β10, Phosphorylated protein kinase B, Vascular endothelial growth factor C

## Abstract

**背景与目的:**

我们前期的研究发现胸腺素β10（thymosin β10, Tβ10）在肺癌中过表达并与肺癌的分期、分化及淋巴结转移呈正相关。本研究旨在探讨外源人重组蛋白Tβ10在肺癌细胞系中促进血管内皮生长因子（vascular endothelial growth factor, VEGF）-C表达情况及其调控机制。

**方法:**

采用RT-PCR法检测不同肺癌细胞系加入外源Tβ10或Tβ10加AKT特异性抑制剂LY294002后VEGF-C mRNA水平的变化；采用Western blot法检测不同肺癌细胞系加入Tβ10或Tβ10加LY294002后VEGF-C及P-AKT蛋白的变化。

**结果:**

在肺癌细胞系SPC-A-1中加入Tβ10可以促进VEGF-C mRNA及蛋白的表达水平，同时促进AKT的磷酸化。在肺癌细胞系A549和LK2中加入Tβ10同样可以促进VEGF-C mRNA及蛋白的表达（*P* < 0.05），并且这种促进作用可以被LY294002所抑制（*P* < 0.05）。

**结论:**

人重组蛋白Tβ10肺癌通过激活AKT的磷酸化促进VEGF-C的表达。

## Abstract (en)

**Background and objective:**

Our previous study found that thymosin β10 overexpressed in lung cancer and positively correlated with differentiation, lymph node metastasis and stage of lung cancer. In this reasearch we aim to study the effects and mechanism of exogenous human recombinant Tβ10 on the expression of VEGF-C on non-small cell lung cancer.

**Methods:**

After SPC, A549 and LK2 cells were treated with 100 ng/mL recombinant human Tβ10, the mRNA level of VEGF-C were detected by RT-PCR. The mean while the protein expression of VEGF-C, P-AKT and AKT were determined by Western blot assay.

**Results:**

Exogenous recombinant human Tβ10 were significantly promote the expression levels of VEGF-C mRNA and protein while promoting the phosphorylation of AKT. Exogenous Tβ10 can promote the expression of VEGF-C mRNA and protein in lung cancer cell lines A549 and LK2 (*P* < 0.05), and this effect can be inhibited by use AKT inhibitor LY294002 (*P* < 0.05).

**Conclusion:**

Tβ10 human recombinant proteins can promote the expression of VEGF-C by activating AKT phosphorylation in lung cancer cell lines.

据《2012年中国肿瘤登记年报》显示：肺癌居全国恶性肿瘤发病率和死亡率第一位。目前，手术治疗仍是治疗肺癌最为行之有效的方法。然而，很多患者在确诊时已经发生了淋巴结转移失去了手术的机会。因此，研究肿瘤淋巴结转移的机制对肺癌的防治有重要意义。肿瘤的淋巴结转移是肿瘤转移的重要途径之一，也是判定肿瘤分期，估计患者预后的重要因素。因此，近年来对于淋巴管特异性标记物及其调控机理的研究逐渐深入，抗肿瘤淋巴管的形成也成为抗肿瘤治疗的新方向。已经证实，血管内皮生长因子（vascular endothelial growth factor, VEGF）-C的表达和淋巴管生成密切相关，在转基因小鼠中过表达VEGF-C能特异性的诱导淋巴内皮细胞增殖和淋巴管的形成^[1]^。VEGF-C是VEGF家族的成员，它可与VEGFR-2和VEGFR-3结合，分别调控血管和淋巴管的生成。

胸腺素是一类分子量5 kDa左右的小分子多肽，最初从小牛胸腺中提取，按等电点不同分为三个家族（α、β和γ）^[2]^。其中胸腺素β10（thymosin β10, Tβ10）是哺乳动物体内含量最为丰富的β族胸腺素之一，研究^[3-5]^证实Tβ10能影响肿瘤细胞的转移和增殖。但目前，Tβ10在肿瘤中的作用存在很大争议。有研究^[4]^发现Tβ10在甲状腺癌、胰腺癌、乳腺癌、结肠癌等肿瘤组织中高表达，同时能够促进肿瘤血管生成和肿瘤细胞侵袭转移，但也有研究^[6]^证明Tβ10在卵巢癌组织和细胞中表达降低，Tβ10过表达可以抑制肿瘤的生长并促进肿瘤的凋亡。然而目前Tβ10在不同肿瘤中所发挥的作用及其机制仍未有定论。

我们课题组前期的研究发现Tβ10在肺癌中表达上调，并与肺癌的分期、分化、淋巴结转移和患者的预后有关^[3]^。这些结果初步证实了Tβ10在肺癌发生发展中所起的促进作用。在本实验中我们课题组将在肺癌细胞系中研究Tβ10对淋巴管标记物VEGF-C的调控作用，并探讨其中的机制。

## 1 材料与方法

### 1.1 主要试剂

人肺癌细胞系SPC-A-1和A549细胞购自ATCC，LK2购自日本癌症研究中心细胞资源库。细胞培养用DMEM高糖培养基购自Gibco，胎牛血清购自碧云天。RNA提取购自Invitrogen，反转录试剂盒、PCR试剂盒及PCR引物合成购自TaKaRa。P-AKT（Ser473）（D9E）单克隆抗体购自CST；AKT、VEGF-C抗体购自Santa Cruz；β-actin抗体购自武汉博士德，辣根过氧化物酶标记的山羊抗小鼠及山羊抗兔IgG购自中杉金桥。BCA法蛋白定量试剂盒购自碧云天，裂解液及超敏发光试剂盒购自Pierce。

### 1.2 细胞培养

SPC-A-1、A549和LK2细胞使用DMEM培养基含10%的灭活小牛血清。37℃、5%CO_2_的条件下培养，每两天换一次液，并用0.25%的胰酶进行传代。实验前取对数生长的细胞，饥饿4 h后根据文献报道和预实验结果（文中未给出）加入100 ng/mL Tβ10，按时间点收集细胞，每次实验同一个处理因素设两个复孔，重复三次实验。

### 1.3 反转录-聚合酶链式反应（reverse transcriptase polymerase chain reaction, RT-PCR）

RT-PCR提取细胞总RNA按RT-PCR（TaKaRa）试剂盒方法进行逆转录反应，反应体系为20 μL，反应结束后再取2 μL的RT产物进行PCR反应，反应体系为20 μL，VEGF-C引物F：5’-TTC CAT TAT TAG ACG TTC CCT G -3’，R：5’-GTG TTT TCA TCA AAT TCT CGG T-3’，片段长度：305 bp，退火温度为54℃；β-actin引物F：5’-AAA TCG TGC GTG ACA TTAA-3’，R：5’-CTC GTC ATA CTC CTG CTT G-3’，片段长度：513 bp，退火温度为55℃。

### 1.4 Western blot法检测蛋白表达

收集细胞并加入裂解液冰浴中裂解，低温高速离心（4℃, 12, 000 rpm, 30 min），提取上清为总蛋白。上清蛋白量为60 μg。12%SDS-PAGE凝胶电泳、转印（60 V, 120 min）到PVDF上。5%BSA室温封闭2 h。一抗分别用P-AKT（1:1, 000）、AKT（1:300）、VEGF-C（1:500）和β-actin（1:500），4 ℃孵育过夜。分别与对应的二抗（1:2, 000）室温孵育2 h，ECL显色，结果经自动电泳凝胶成像分析仪采集。进行灰度测定。

### 1.5 统计学方法

使用SPSS 16.0统计软件，两组之间用*t*检验进行数据分析，*P* < 0.05为差异有意义。

## 2 结果

### 2.1 Tβ10促进SPC-A-1细胞中VEGF-C mRNA表达

取对数生长的SPC-A-1细胞接种于六孔板中，待其贴壁后在实验组加入浓度为100 ng/mL的人重组Tβ10，对照组加入同体积的PBS。分别在0 h、1 h、2 h、4 h、8h、24 h收集细胞检测各组VEGF-C mRNA的水平。结果发现，加入Tβ10后细胞内VEGF-C mRNA含量逐渐上升（图 1）。

**1:**
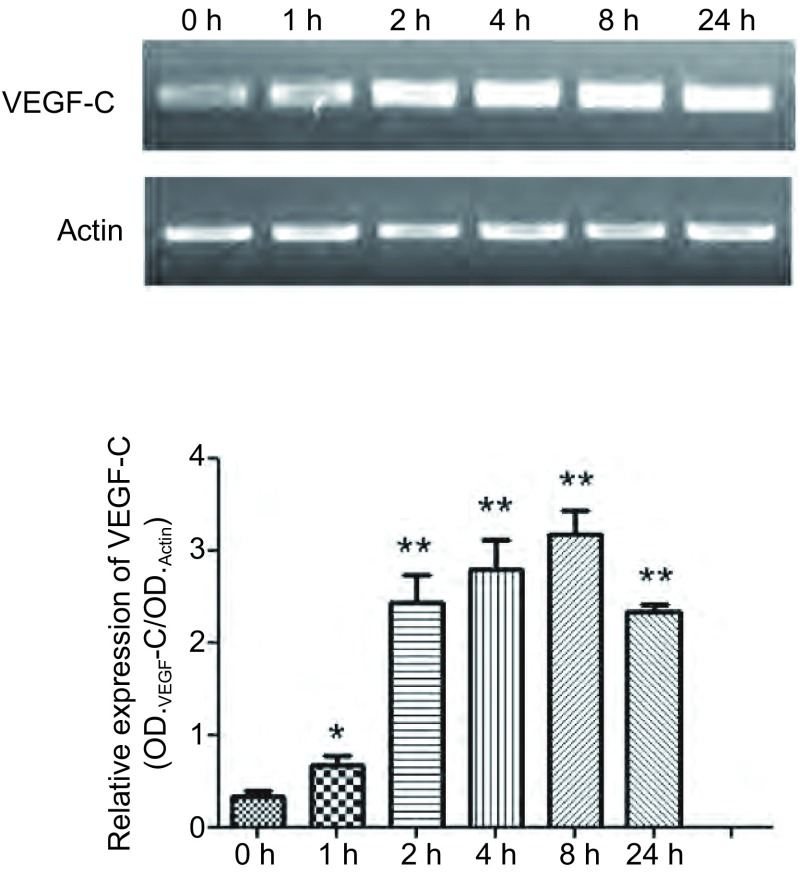
SPC-A-1细胞系中加入100 ng/mL人重组T*β*10后0 h、1 h、2 h、4 h、8 h、24 h，用RT-PCR的方法检测细胞内VEGF-C mRNA变化，柱图显示加入T*β*10后SPC-A-1细胞中VEGF-C mRNA水平随时间推移逐渐上升。^*^：与对照组相比，*P* < 0.05；^**^：与对照组相比，*P* < 0.01。 After adding 100 ng/mL human recombinant T*β*10 in SPC-A-1, cells were collected in 0 h、1 h、2 h、4 h、8 h、24 h, then RT-PCR was used to detected the mRNA level of VEGF-C. Bar graph display after adding T*β*10, mRNA levels of VEGF-C in SPC-A-1 cells gradually increased over time. ^*^: Compared with the control, *P* < 0.05; ^**^: Compared with the control, *P* < 0.01.

### 2.2 在SPC-A-1细胞中Tβ10能够促进P-AKT及VEGF-C蛋白表达

在SPC-A-1细胞中加入Tβ10后分别在0 h、1 h、2 h、4 h、8 h、24 h收集细胞，提取总蛋白后检测不同组细胞P-AKT、AKT及VEGF-C蛋白变化。我们发现加入浓度为100 ng/mL的Tβ10后P-AKT及VEGF-C蛋白水平随时间的延长逐渐升高（*P* < 0.05），但是总的AKT水平未见明显变化（图 2）。这些结果说明在SPC-A-1细胞中Tβ10可能是通过促进AKT磷酸化激活促进VEGF-C表达的。

**2:**
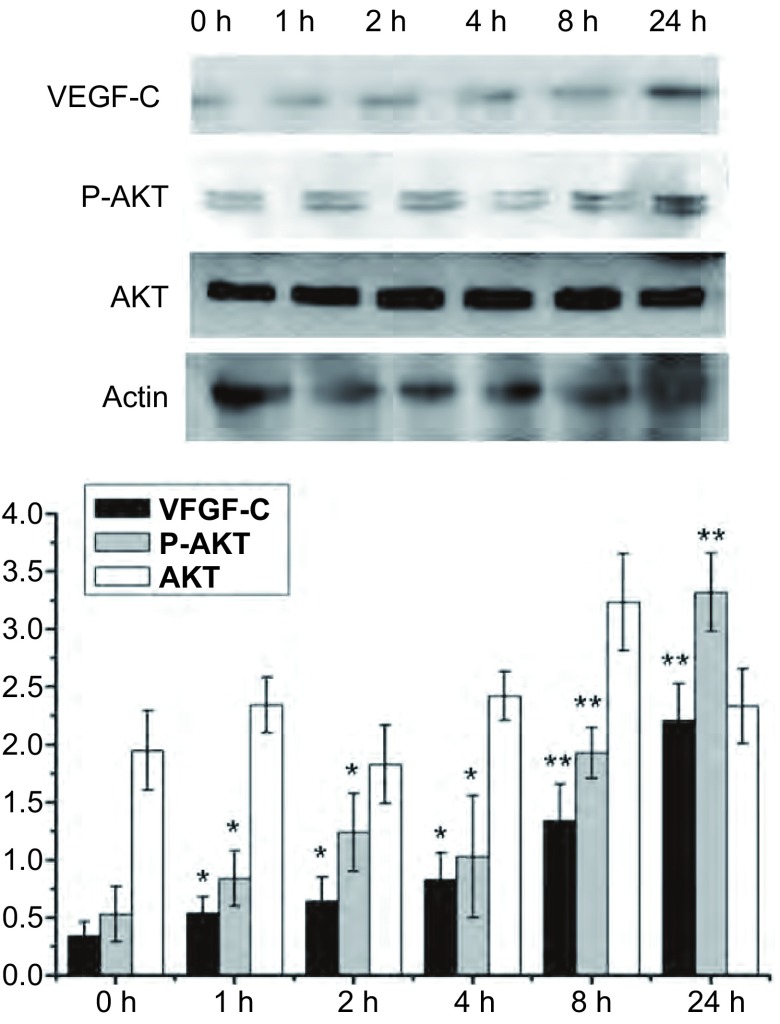
SPC-A-1细胞系中加入100 ng/mL人重组T*β*10后0 h、1 h、2 h、4 h、8 h、24 h，用Western blot检测细胞内VEGF-C、P-AKT和AKT蛋白变化。如图所示，VEGF-C和P-AKT蛋白水平逐渐升高，而总AKT水平不变。^*^：与对照组相比，*P* < 0.05；^**^：与对照组相比，*P* < 0.01。 After adding 100 ng/mL human recombinant T*β*10 in SPC-A-1, cells were collected in 0 h、1 h、2 h、4 h、8 h、24 h, then Western blot was used to detected the protein level of VEGF-C, P-AKT and AKT. As the figure show, the protein level of VEGF-C and P-AKT were increased gradually, but the total AKT level were unchanged. ^*^: Compared with the control, *P* < 0.05; ^**^: Compared with the control, *P* < 0.01.

### 2.3 在SPC-A-1细胞中Tβ10可能通过促进P-AKT水平促进VEGF-C mRNA表达

在SPC-A-1细胞中加入Tβ10，8 h后在一组细胞中加入P-AKT特异性抑制剂LY294002，另一组细胞不加。加入抑制剂0 h、1 h、2 h、4 h、8 h、24 h后分别提取细胞的mRNA，检测其中VEGF-C水平。结果发现，随着时间的进展，不加抑制剂组VEGF-C的水平变化很少，而加LY294002组VEGF-C mRNA含量逐渐下降，说明LY294002能明显抑制VEGF-C表达（*P* < 0.05）（图 3）。

**3:**
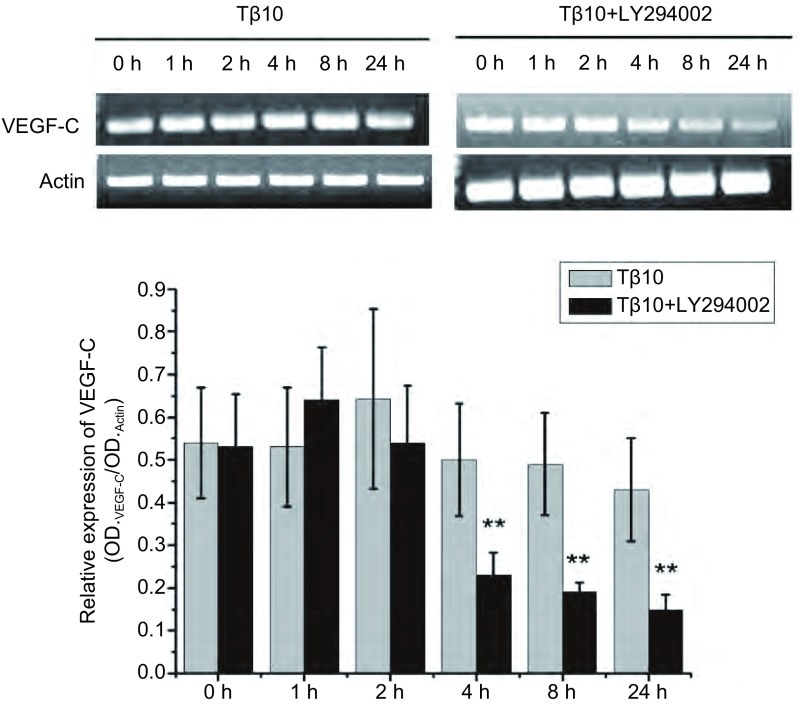
在SPC-A-1细胞中加入T*β*10 8 h后一组加入LY294002, 另一组不加，0 h、1 h、2 h、4 h、8 h、24 h后细胞的VEGF-C mRNA变化比较，发现LY294002可明显抑制T*β*10引起的VEGF-C升高。^**^：与对照组相比，*P* < 0.01。 8 h after adding T*β*10 in SPC-A-1 cells, LY294002 was added in the medium. After 0 h、1 h、2 h、4 h、8 h、24 h, mRNA levels of VEGF-C were detected. The results reveals that LY294002 significantly inhibited T*β*10 induced VEGF-C increased. ^**^: Compared with the control, *P* < 0.01.s

### 2.4 在不同细胞系中Tβ10通过上调P-AKT水平促进VEGF-C表达

为了进一步验证Tβ10促进VEGF-C表达的结论，我们又选择了两个细胞系：A549及LK2分别加入Tβ10和LY294002，观察细胞中VEGF-C蛋白及mRNA变化。在两种细胞系中加入Tβ10可以促进VEGF-C蛋白及mRNA上调，如果在加入Tβ10的同时加入AKT通路特异性抑制剂LY294002则会使VEGF-C蛋白及mRNA水平下调（*P* < 0.01）（图 4，图 5）。可见，在肺癌细胞系中Tβ10通过上调P-AKT水平促进VEGF-C表达。

**4:**
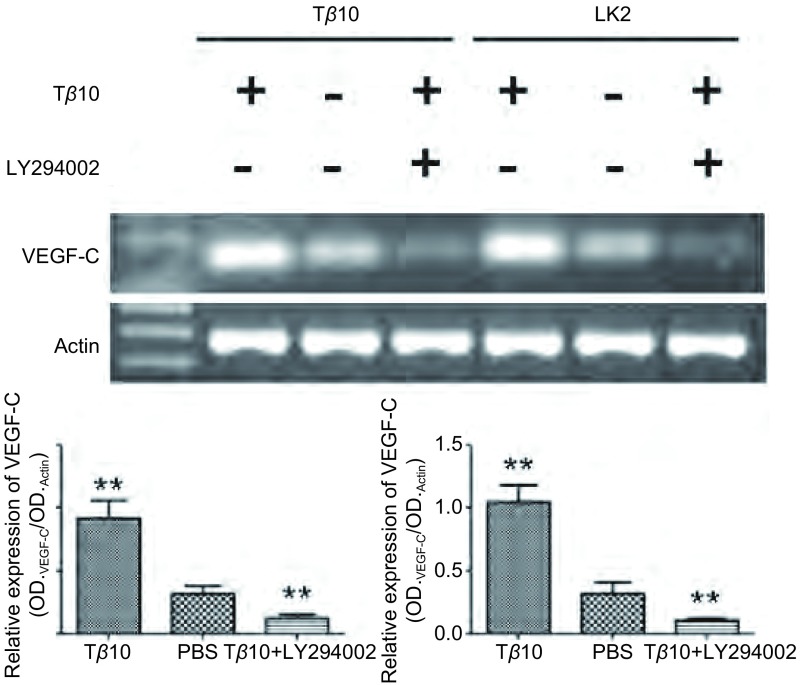
在A549和LK2细胞中加入T*β*10和LY294002，检测细胞VEGF-C mRNA表达变化，发现T*β*10能促进这两株细胞VEGF-C mRNA的表达，LY294002则能逆转该变化。^**^：与对照组相比，*P* < 0.01。 In A549 and LK2 cells after adding T*β*10 and LY294002, the mRNA level of VEGF-C was detected. T*β*10 can prompted the expression of VEGF-C, and LY294002 can inhibited the expression of VEGF-C. ^**^: Compared with the control, *P* < 0.01.

**5:**
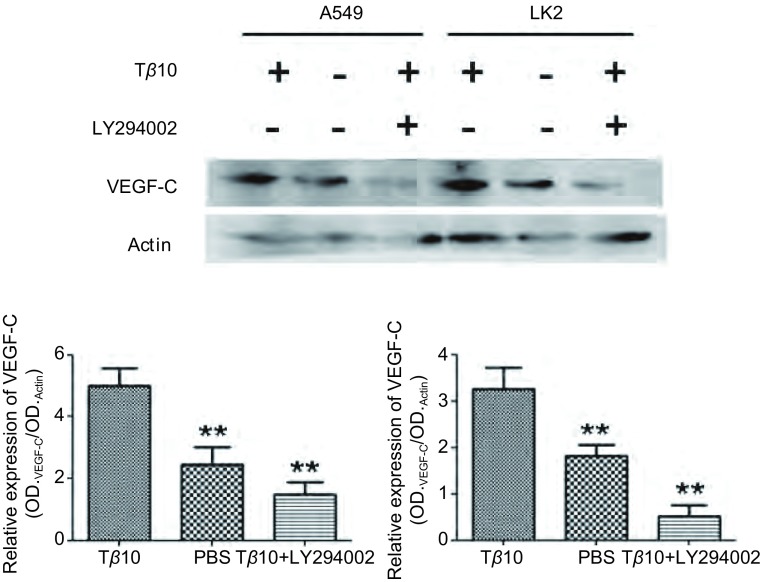
在A549和LK2细胞中加入T*β*10和LY294002，检测细胞VEGF-C蛋白表达变化，发现T*β*10能促进这两株细胞VEGF-C蛋白表达，LY294002则能逆转这种变化。^**^：与对照组相比，*P* < 0.01。 In A549 and LK2 cells after adding T*β*10 and LY294002, the protein level of VEGF-C was detected. T*β*10 can prompted the expression of VEGF-C, and LY294002 can inhibited the expression of VEGF-C. ^**^: Compared with the control, *P* < 0.01.

## 3 讨论

近年来，胸腺素类家族的小分子化合物在肿瘤中的作用受到了越来越广泛的关注，在许多肿瘤中都发现了胸腺素的高表达。Tβ4在肾癌、肺癌、结肠癌、乳腺癌中表达增高^[7, 8]^，而且Tβ4的高表达可能直接增加肿瘤转移的机会。Tβ10在乳腺癌、黑色素瘤、生殖细胞肿瘤、肺癌等中表达升高^[9, 10]^。Tβ15则在前列腺癌、乳腺癌^[11]^中高表达。但也有报道^[5, 6]^某些胸腺素在肿瘤中低表达甚至缺失。这些研究表明不同种类的胸腺素在不同组织中的作用机制存在很大差异，这就需要更深入的实验来验证胸腺素类的生理作用。

肿瘤淋巴管生成主要受VEGF-C、-D和VEGFR-3调控。近年来多数研究报道称：恶性肿瘤的淋巴结转移是因为恶性肿瘤细胞产生大量VEGF-C，并通过下述3种途径参与调节淋巴结转移：①VEGF-C通过与VEGFR-3受体结合，刺激诱导癌旁淋巴管增生，并降低淋巴管内皮细胞间的粘附，增加了肿瘤淋巴结转移机会^[12]^；②提高肿瘤细胞粘附作用。能分泌VEGF-C的肿瘤细胞通过淋巴管内皮细胞上受体的亲和作用，使肿瘤细胞得以粘附到淋巴管内皮细胞上；③VEGF-C还可作为一种趋化因子，使肿瘤细胞向淋巴管方向迁移。

我们课题组前期的研究发现，Tβ10在肺癌中表达上调并与肺癌淋巴管密度及VEGF-C表达密切相关，这促使我们进一步研究Tβ10在肺癌中通过上调VEGF-C表达促进淋巴管形成的机制。AKT和肿瘤血管及淋巴管生成密切相关，Tsutsui等^[13]^证明在乳腺癌中AKT可以通过VEGF-C促进淋巴管形成。而另有文献^[7]^报道与Tβ10同家族的Tβ4可以在内皮细胞中促进AKT磷酸化从而发挥Tβ4促进细胞运动的功能。因此我们推测Tβ10在肺癌细胞中可能通过促进AKT磷酸化促进VEGF-C的表达。

由于胸腺素是小分子多肽，根据文献报道可以采用外源加入人重组蛋白的方法检测胸腺素类的生理作用^[7, 14]^，因此在本实验中我们采用的是加入外源Tβ10的实验方法。研究结果发现，在SPC-A-1细胞中加入100 ng/mL人重组Tβ10可以促进VEGF-C mRNA和蛋白表达水平。为了进一步验证Tβ10是否是通过促进AKT磷酸化促进VEGF-C表达，我们又同时检测了加入Tβ10后P-AKT的表达情况，发现加入Tβ10后在VEGF-C mRNA和蛋白表达水平上调的同时P-AKT表达也升高，而总的AKT的水平不发生变化。如果在加入Tβ10同时又加入了P-AKT抑制剂则能明显抑制VEGF-C表达。为了能证明我们推测的结论，我们又在另外两株肺癌细胞系中重复了实验，发现在不同细胞系中Tβ10均能通过P-AKT通路促进VEGF-C表达。这些实验均说明在肺癌中Tβ10促进淋巴管生成的功能是通过促进AKT磷酸化进而促进VEGF-C表达来实现的。

综上所述，Tβ10在肺癌中促进淋巴管形成的作用是通过激活AKT通路促进VEGF-C表达实现的。Tβ10在肺癌中作为一个癌基因，对它的深入研究对阐明肺癌的发生发展机制，尤其是肿瘤淋巴结转移机制有着重要的意义。

## References

[1] Jeltsch M, Kaipainen A, Joukov V (1997). Hyperplasia of lymphatic vessels in VEGF-C transgenic mice. Science.

[2] Domanski M, Hertzog M, Coutant J (2004). Coupling of folding and binding of thymosin beta 4 upon interaction with monomeric actin monitored by nuclear magnetic resonance. J Biol Chem.

[3] Gu Y, Wang C, Wang Y (2009). Expression of thymosin beta 10 and its role in non-small cell lung cancer. Hum Pathol.

[4] Li M, Zhang Y, Zhai Q (2009). Thymosin beta-10 is aberrantly expressed in pancreatic cancer and induces JNK activation. Cancer Invest.

[5] Kim YC, Kim BG, Lee JH (2012). Thymosin beta 10 expression driven by the human TERT promoter induces ovarian cancer-specific apoptosis through ROS production. PLoS One.

[6] Lee SH, Zhang W, Choi JJ (2001). Overexpression of the thymosin beta-10 gene in human ovarian cancer cells disrupts F-actin stress fiber and leads to apoptosis. Oncogene.

[7] Maelan AE, Rasmussen TK, Larsson LI (2007). Localization of thymosin beta 10 in breast cancer cells: relationship to actin cytoskeletal remodeling and cell motility. Histochem Cell Biol.

[8] Rouzaut A, Irigoyen M, Montuenga LM (2007). Lymphangiogenesis and lung cancer. J Thorac Oncol.

[9] Nemolato S, Ekstrom J, Cabras T (2013). Immunoreactivity for thymosin beta 4 and thymosin beta 10 in the adult rat oro-gastro-intestinal tract. Eur J Histochem.

[10] Feher LZ, Pocsay G, Krenacs L (2012). Amplification of thymosin beta 10 and *AKAP13* genes in metastatic and aggressive papillary thyroid carcinomas. Pathol Oncol Res.

[11] Darb-Esfahani S, Kronenwett R, von Minckwitz G (2012). Thymosin beta 15A (™SB15A) is a predictor of chemotherapy response in triple-negative breast cancer. Br J Cancer.

[12] Joukov V, Pajusola K, Kaipainen A (1996). A novel vascular endothelial growth factor, VEGF-C, is a ligand for the Flt4 (VEGFR-3) and KDR (VEGFR-2) receptor tyrosine kinases. EMBO J.

[13] Tsutsui S, Matsuyama A, Yamamoto M (2010). The Akt expression correlates with the VEGF-A and -C expression as well as the microvessel and lymphatic vessel density in breast cancer. Oncol Rep.

[14] Huang WQ, Wang BH, Wang QR (2006). Thymosin beta 4 and AcSDKP inhibit the proliferation of HL-60 cells and induce their differentiation and apoptosis. Cell Biol Int.

